# Is There Any Purpose in Routine Syndesmotic Screw Removal? Systematic Literature Review

**DOI:** 10.3390/jcm13164805

**Published:** 2024-08-15

**Authors:** Błażej G. Wójtowicz, Katarzyna Chawrylak, Jędrzej Lesman, Marcin Domżalski

**Affiliations:** 1 Department of Orthopedics and Traumatology of the Musculoskeletal System, WAM University Clinical Hospital, Central Veterans Hospital, Żeromskiego 113 St., 90-549 Lodz, Poland; jedreklesman@yahoo.pl (J.L.); marcin.domzalski@umed.lodz.pl (M.D.); 2 Department of Surgical Oncology, Medical University of Lublin, Radziwiłłowska 13 St., 20-080 Lublin, Poland; 56646@student.umlub.pl

**Keywords:** syndesmosis, syndesmotic screw, malleoli fracture, tibiofibular syndesmosis

## Abstract

**Introduction**: The aim of this systematic review is to examine the recent evidence comparing the removal and non-removal of syndesmotic screws in tibiofibular syndesmosis injuries in terms of functional, clinical, and radiographic outcomes. **Methods**: A comprehensive literature review was conducted to identify clinical studies on syndesmotic screw removal and its outcomes, searching the Cochrane Library and PubMed Medline for publications from 1 January 2004 to 12 February 2024. Studies were included if they involved tibiofibular syndesmotic screw fixation, assessed screw removal or retention, described clinical outcomes, and were original research with at least fifteen patients per group. **Results**: Most reviewed articles (18 out of 27; 67%) found no significant differences between the routine removal and retention of syndesmotic screws post-fixation. Four retrospective studies (15%) suggested that retaining screws might result in worse outcomes compared to removal. Two studies (7%) indicated that removing screws could introduce additional risks. One study (4%) observed that post-removal, there is some fibula–tibia separation without affecting the medial clear space. Another study (4%) noted that intraosseous screw breakage might increase the need for implant removal due to pain. Additionally, no significant differences in ankle function were found among groups with varying intervals of screw removal. **Conclusions**: The current literature does not definitively support routine removal of syndesmotic screws. Given the potential complications and financial costs, routine removal should not be performed unless specifically indicated.

## 1. Introduction

Malleoli fractures are one of the most common orthopedic injuries. Associated injuries to the tibiofibular syndesmosis may account for approximately 20% of cases [1]. It is believed that the tibiofibular syndesmosis heals after two to three months, and the screw that fixes the syndesmosis is not needed after this period [2]. From a biomechanical point of view, screw fixation is associated with issues in restoring fibular rotation, leading to increased distal tibiofibular space, which can limit ankle mobility [3]. Additionally, anatomical factors such as limited preinjury range of motion in dorsiflexion can impact the degree of mobility limitation when using syndesmotic screws [4]. The timeframe for these limitations can vary, but studies indicate that they may persist at least until the follow-up periods, which are typically around two years postoperation [3,4]. There are differing opinions on the optimal number of screws or the use of suture-buttons, and how many cortical layers the screws should traverse to effectively stabilize the syndesmosis [3]. Recent studies suggest that suture-button fixation provides superior early postoperative outcomes compared to traditional screw fixation, although no long-term superiority has been definitively established. The choice between these methods often depends on individual case assessments and surgeon preference. Screw fixation traditionally involves the screw passing through three or four cortices, but the precise protocol can vary. The suture-button technique, on the other hand, generally does not require crossing as many cortical layers and tends to involve fewer complications related to implant removal [3]. There is also no clear opinion on whether the syndesmotic screw should be removed routinely and, if so, when is the best time for removal. Therefore, the decision to remove the syndesmotic screw is often based on the surgeon’s own beliefs [5,6,7,8,9]. In the past, several studies have evaluated the routine removal of syndesmotic screws (Table 1) [10,11]. Most of them showed no significant difference in results between the screws retained or removed. The aim of this systematic review is to examine the recent evidence comparing the removal and non-removal of syndesmotic screws in tibiofibular syndesmosis injuries in terms of functional, clinical, and radiographic outcomes.

## 2. Materials and Methods

### 2.1. Search Strategy

A comprehensive literature review was conducted to identify clinical studies investigating syndesmotic screw removal and assessing patients’ clinical, radiographic, or functional outcomes. The search was carried out in the Cochrane Library and PubMed Medline electronic databases, covering studies published from 1 January 2004 to 12 February 2024. The search terms used included “syndesmosis” OR “syndesmotic” OR “transsyndesmotic” OR “distal tibiofibular” AND “screw” AND “remove”. The publication date range was chosen to ensure the retrieval of the most recent findings on syndesmotic screw removal in ankle fracture patients. Articles that reported on postoperative outcomes following syndesmotic screw fixation, irrespective of screw number, size, or position, were considered eligible for inclusion.

### 2.2. Selection

The first criterion was that studies must have been published within the past 20 years. This criterion was set to avoid outdated data that might not reflect current medical practices and understandings. Studies published more than 20 years ago were excluded.

The next step involved verifying that the study involved tibiofibular syndesmotic screw fixation. This ensured that the procedure in question was relevant to the research focus. Studies that did not involve tibiofibular syndesmotic screw fixation were excluded.

The researchers then assessed whether the study included patients where the syndesmotic screw was either removed or retained. This was crucial to focus on the specific intervention being studied. Studies where neither removal nor retention of the syndesmotic screw was assessed were excluded.

The study was required to describe the outcomes of the intervention, including clinically important results such as physical examinations, measurements, and complications. This ensured that the study provided useful and detailed outcomes for the analysis. Studies that did not describe the outcomes of the screw removal or retention were excluded.

It was necessary for the study to be an original study with a prospective or retrospective comparative design, excluding case series and meta-analyses. This criterion ensured high methodological quality. Case series were excluded due to their lower level of scientific evidence and higher risk of bias, often involving only a few individuals. The review focused on studies with at least 15 patients in each group, with most studies considering over 50 patients in total, making case series less significant.

Meta-analyses were excluded because they often included the same original studies already in this review, leading to the duplication of results and potential distortion of data interpretation. Ensuring a minimum of fifteen patients in each group provided a sufficient sample size for reliable results. Additionally, studies performed on the same group of patients at different times were excluded to avoid duplicative data that might skew the analysis.

Inclusion Criteria:

- Studies published within the past 20 years.
- Studies involving tibiofibular syndesmotic screw fixation.
- Studies assessing the intervention of the removal or retention of the syndesmotic screw.
- Studies describing outcomes of the intervention, including clinically important results.
- Original studies with a prospective or retrospective comparative design.
- Studies with a minimum of fifteen patients in each group.

Exclusion Criteria:

- Studies older than 20 years.
- Case series, reviews, or meta-analyses.
- Studies with fewer than fifteen patients in each group.
- Studies involving the same group of patients at different times.

### 2.3. Assessment of Quality

One reviewer (B.W.) assessed the methodological quality of each included study in terms of study design, type of intervention, follow-up time, and similarity of surgical procedures. A study was considered to be prospective if it started before the first patient was enrolled. In contrast, a study was considered to be retrospective if it started after the first patient was enrolled.

### 2.4. Data Extraction

Specific data extracted from the research were recorded in sheets. The specific data extracted included the country in which the study was primarily conducted, study duration, number of eligible patients, type of surgical intervention, number of patients who underwent syndesmotic screw removal, patient-oriented outcomes, and scales. These worksheets were subsequently compared, and any discrepancies were resolved through a review of the original study and discussions to achieve consensus.

## 3. Results

**Figure 1:**
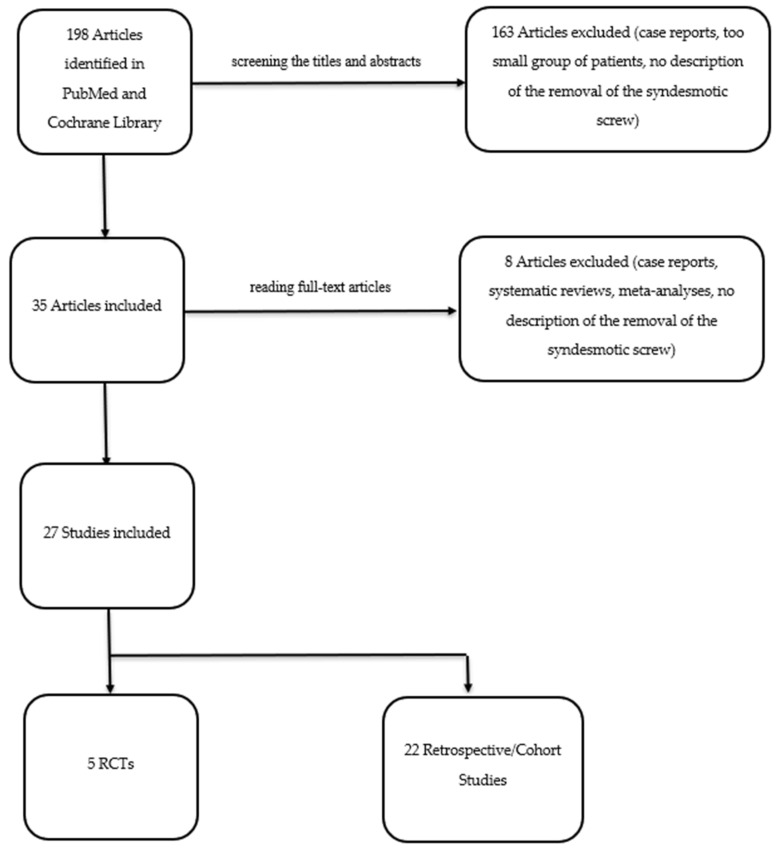
Search strategy flow chart according to PRISMA checklist. RCT—randomized controlled trial.

A total of 198 articles were retrieved through the search process. After screening the titles and abstracts, 163 articles were excluded. Among the remaining 35 articles, an additional 8 were eliminated after reading the full text. These exclusions were based on the articles being either case reports [12], systematic reviews [5,6,7,9], meta-analyses [8], or studies in which syndesmotic screws were not removed. Of the twenty-seven articles that remained, five were identified as randomized controlled trials (RCTs), while the remaining twenty-two studies were retrospective or cohort studies (see Figure 1).

The findings from both the RCTs and non-randomized investigations are outlined in Table 2. 

**Figure 2:**
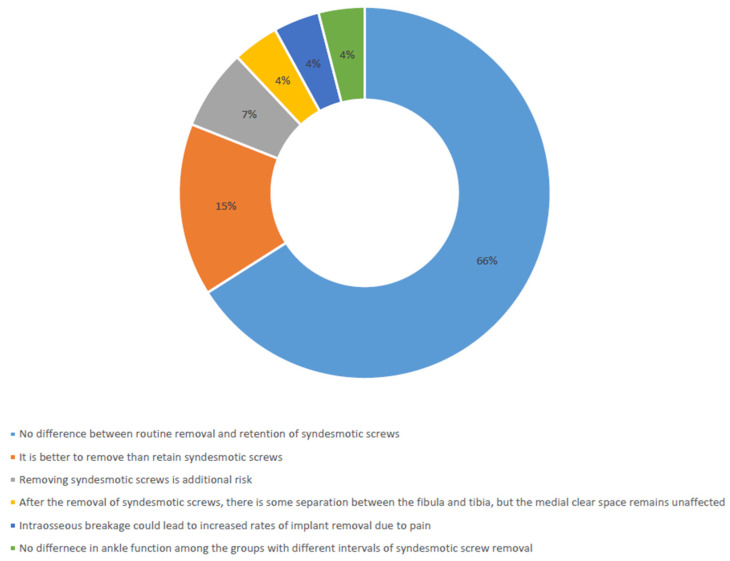
Summary conclusions from reviewed studies.

The majority of these articles indicated no significant variance between the routine removal and retention of syndesmotic screws following tibiofibular syndesmosis fixation (18 out of 27; 67%) [13,14,15,16,17,18,19,20,21,22,23,24,25,26,27,28,29,30]. Only four retrospective studies suggested that retaining syndesmotic screws might yield inferior outcomes compared to their removal (4 out of 27; 15%) [31,32,33,34]. Additionally, some studies suggested that removing syndesmotic screws could pose additional risks for patients (2 out of 27; 7%) [35,36]. After the removal of syndesmotic screws, there is some separation between the fibula and tibia, but the medial clear space remains unaffected (1/27 cases; 4%) [37]. It appears that syndesmotic screw breakage might pose greater challenges than previously understood. In particular, intraosseous breakage could lead to increased rates of implant removal due to pain (1/27 cases; 4%) [38]. Additionally, there are no notable discrepancies in ankle function among the groups with different intervals of syndesmotic screw removal (1/27 cases; 4%) (Figure 2) [39]. A meta-analysis was considered impossible due to the heterogeneity of the data.

**Table 2:** Characteristics of studies included in this review. AAOS—American Academy of Orthopedic Surgeons Scale; AOFAS—American Orthopaedic Foot and Ankle Society Scale; AP—Anteroposterior; CRPS—Complex Regional Pain Syndrome; CS—Clear Space; CT—Computed Tomography; EQ-5D—EuroQol-5 Dimension; FAOS—Foot and Ankle Outcome Score; i.v.—In Venous; IQR—Interquartile Range; MCS—Medial Clear Space; MO—Ankle Mortise Radiograph; ODR—On-Demand Removal; OL—Tibia–Fibula Overlap; OMAS—Olerud–Molander Scale; RODEO—Routine versus On-Demand Removal of the Syndesmotic Screw; RR—Routine Removal; SD—Standard Deviation; SF12-MCS—Short-Form-12 Mental Component Summary; SF12-PCS—Short-Form-12 Physical Component Summary; SMFA—Short Musculoskeletal Function Assessment; SSI—Surgical Site Infection; VAS—Visual Analog Scale.

Author, Year of the Study	Type of Study	Strength of Evidence	Number of Patients	Number of Participants Undergoing Screw Removal	Main Outcomes	Complications	Specific Findings
Andersen 2015 [35]	Retrospective Cohort	4	161	161	Incidence of complications following routine syndesmotic screw removal: 6%.	Wound infection in 8 (5%) patients. Serious infections requiring hospitalization and intravenous antibiotics: 3 patients. Surgical revisions needed: 2 patients. Treated by oral antibiotics: 5 patients. *Staphylococcus aureus* identified in 6/8 cases with positive culture. Postoperative infection associated with more pain (5.3 vs. 2.3; *p* = 0.02). Postoperative infection associated with lower satisfaction (4.7 vs. 7.6; *p* = 0.014).	Data do not support routine removal. Recommend routine antibiotic prophylaxis if removal chosen—single dose Cefalotin at 2 g i.v.
Bell 2006 [13]	Retrospective Cohort	4	30	23	No statistically significant variance in ankle scores between groups. Occurrence of screw breakage consistent in retained group after six months. Osteolysis occurrence consistent in retained group after six months.	Complications in five patients postoperatively. Syndesmotic screw removed: malposition of medial malleolar screw in one patient, requiring repositioning after 2 days. Syndesmotic screw removed: superficial wound infection in one patient, resolving on oral antibiotics after 1 week. Syndesmotic screws left in situ: superficial wound infection in one patient, resolving on oral antibiotics after 2 weeks.	Functional outcomes similar: ankle scores of 88 ± 5.50 (screws removed) vs. 86 ± 7.46 (screws retained). No significant difference in ankle scores (*p* = 0.79). Pain-free walking: 11/23 (48%) with screws removed, 2/7 (29%) with screws retained (*p* > 0.05). Return to work without restrictions: 13/23 (57%) with screws removed, 4/7 (57%) with screws retained (*p* > 0.05). No significant difference in range of motion deficit between groups in flexion/extension and inversion/eversion (*p* > 0.05).
Boyle 2014 [14]	Randomized Controlled Trial	1	51	26	No statistically significant difference in ankle scores. Persistent screw breakage in the retained group after six months. Ongoing osteolysis in the retained group after six months.	No intra-operative complications. Recurrent diastasis and broken syndesmotic screw in one patient (screw retention group) four months postoperatively, managed with revision surgery. Wound infection in two patients (screw removal group) after screw removal.	Mean removal time: 116 days (range: 81 to 177). No significant effect of removal time on OMAS (*p* = 0.507). AOFAS ankle–hindfoot score (*p* = 0.860). AAOS foot and ankle score (*p* = 0.818). Pain VAS (*p* = 0.596). Ankle dorsiflexion (*p* = 0.818). Ankle plantarflexion (*p* = 0.911). Calf girth (*p* = 0.221). Tibiofibular clear space (*p* = 0.279).
Egol 2010 [15]	Retrospective Cohort	4	79	11	No statistical differences in outcomes between Removed screws and intact screws. Broken screws and intact screws.	Asymptomatic nonunion of medial malleolus. Symptomatic fibular nonunion, treated with secondary plating and bone graft. Delayed fibular union. Three draining wounds, treated with antibiotics. One arthrodesis after early deep infection. No differences in complication rates between syndesmotic fixation and non-fixation groups.	Comparison of screws: failed/removed (n = 26) vs. intact (n = 53). No statistical difference in Pain (*p* = 0.87). Function (*p* = 0.82). Range of ankle motion (*p* = 0.20). Comparison of failed/removed screws (n = 26) vs. no syndesmotic injury (n = 268): More no pain reports in the no-syndesmotic-injury group (*p* = 0.049). Trend towards greater function in no-syndesmotic-injury group (*p* = 0.068).
Gennis 2015 [16]	Retrospective Cohort	4	166	58	No statistically significant difference in radiographic displacement outcomes: Syndesmosis. Mortise Comparison: screw removal vs. intact or broken screws.	No data.	Removal of syndesmotic screws at 3 months: Slightly lower OL (< 1 mm) on mortise radiographs. Greater CS (0.5 mm) on mortise radiographs. No talar subluxation observed. Differences were not statistically significant. Mortise remained intact regardless of screw status: Removed. Loosened or broken. Retained solid.
Hamid 2009 [17]	Retrospective Cohort	4	52	15	No statistical difference in clinical outcomes: Syndesmosis screw removal vs. no removal. Broken screws appeared to have the most favorable outcomes.	Two patients (5%) with retained screws had local tenderness.	Mean VAS: 2.02 (SD: 2.70) in screw-retained group vs. 0.074 (SD: 0.97) in screw-removed group (*p* = 0.268). Mean AOFAS score: 85.59 (SD: 13.83) in screw-retained group vs. 85.80 (SD: 11.33) in screw-removed group (*p* = 0.714).
Huevel 2023 [18]	Randomized Controlled Trial	1	109	77 (24 on demand)	No functional difference between on-demand and routine removal for syndesmotic injuries during four-year follow-up. Findings corroborate the primary RODEO trial. On-demand removal should be considered standard practice following syndesmotic screw fixation.	Pain: 7 patients. Stiffness: 2 patients. Broken or loosened screw: 3 patients. Desire for removal without further explanation: 3 patients. Combination of complications: 9 patients.	Median OMAS score: RR group: 85.0. ODR group: 90.0 (*p* = 0.384). Secondary outcome measures: AOFAS: RR group: 88.0. ODR group: 90.0 (*p* = 0.722). FAOS: RR group: 87.5. ODR group: 92.9 (*p* = 0.399). EQ-5D: RR group: 0.87. ODR group: 0.96 (*p* = 0.092).
Hoines 2004 [19]	Randomized Controlled Trial	1	64	32 (2 on demand)	No difference in functional outcomes between single-quadcortical-screw-removal group and two-tricortical-screw-retention group.	Three tricortical syndesmosis screws removed in 2 patients due to spontaneous loosening and telescoping after 3 months. High incidence of deep surgical site infections (6.3%) with biodegradable screws considered coincidental and unrelated to fixation method.	OMAS at 3 months: Tricortical group: 77 points. Quadricortical group: 66 points (*p* = 0.025). OMAS after 1 year: Tricortical group: 92.6 points. Quadricortical group: 85.7 points (*p* = 0.192). Pain significantly lower in tricortical group after 3 months (*p*= 0.017). No significant difference in pain after 1 year.
Hsu 2011 [39]	Retrospective Cohort	4	52	52	No notable differences in ankle function across groups with varying intervals of syndesmotic screw removal.	Syndesmotic screw breakage: Within three months: 3 patients (15.0%) in group 2. Beyond three months: 2 patients (15.4%) in group 3 (at 6 and 12 months). Group 1: No screw breakage. Statistically significant overall rate of screw breakage among the three groups (*p* = 0.034). No significant difference in breakage between Group 1 and group 2 (*p* = 0.125). Group 1 and group 3 (*p* = 0.157). Group 2 and group 3 (*p* = 0.375).	No statistical difference in ankle function among the four groups (*p* = 0.051). No significant difference in ankle function between syndesmotic diastasis with or without associated ankle fractures (*p* = 0.410). No significant difference in ankle function among the three groups with different intervals of syndesmotic screw removal (*p* = 0.191). No significant difference in ankle function between patients with or without syndesmotic screw breakage (*p* = 0.343). No significant difference in ankle function between patients with or without syndesmotic diastasis recurrence (*p* = 0.218).
Huang 2022 [20]	Retrospective Cohort	4	63	63	Diastasis observed at end of follow-up after syndesmotic screw removal. Diastasis developed prior to screw removal, not as a result of it.	All included patients followed similar rehabilitation protocols. No major complications or dropouts.	OL decreased by an average of 2.0 mm ± 2.8 mm (range: −10.1 to 5.0 mm; *p* < 0.001). CS increased by an average of 0.8 mm ± 1.3 mm (range: −1.8 to 5.8 mm; *p* < 0.001). MCS increased by an average of 0.1 mm ± 1.3 mm (range: −2.8 to 3.6 mm; *p* = 0.495). Significant changes in OL and CS; no significant change in MCS.
Ibrahim 2022 [38]	Retrospective Cohort	4	43	21	Syndesmotic screw breakage is more challenging than previously acknowledged. Intraosseous breakage may be linked to increased implant removals due to pain. Placing screws at least 20 mm above the tibiotalar joint might reduce intraosseous breakage risk. Increased screw placement height could minimize the need for implant removal postoperatively.	No data.	Screws placed further from the tibiotalar joint had a lower risk of intraosseous breakage (OR: 0.818, *p* = 0.002). Screws placed at a height of 20 mm or greater were more likely to break in the clear space (OR: 12.1, *p* = 0.002). No significant association between breakage location and screw diameter, length, or angulation.
Juarez-Jimenez 2018 [36]	Retrospective Cohort	4	207	207	Frequency of complications associated with syndesmotic screw removal was lower in this study compared to existing literature.	Five patients with complications observed (2.41%). Wound dehiscence: 2 cases. Superficial infection: 2 cases (1.92%). Subsequent diastasis of the syndesmosis with pain due to instability: 1 case (0.48%).	Prevalence of complications related to syndesmotic screw removal in our hospital was lower than reported in the surgical literature. Syndesmotic screw removal is considered a safe procedure with low risk for infection and post-traumatic ankle instability.
Jordan 2011 [37]	Retrospective Cohort	4	86	86	Diastasis of the fibula from the tibia observed after transsyndesmotic screw removal. Medial clear space remained unchanged.	No data.	CS: Pre-screw removal (12.5 weeks): AP view: 4.63 mm (SD ± 1.62). MO view: 4.73 mm (SD ± 1.58). Post-screw removal: AP view: 5.41 mm (SD ± 1.67). MO view: 5.53 mm (SD ± 1.47). Statistically significant increase in both views (*p* ≤ 0.000). MCS: Pre-screw removal (12.5 weeks): AP view: 2.84 mm (SD ± 0.64). MO view: 2.99 mm (SD ± 0.61). Post-screw removal: AP view: 2.97 mm (SD ± 0.67). MO view: 3.05 mm (SD ± 0.66). Significant increase in AP view (*p* = 0.034); no significant change in MO view. Overlap of the lateral (OL): Pre-screw removal (12.5 weeks): AP view: 5.83 mm (SD ± 2.54). MO view: 2.29 mm (SD ± 2.17). Post-screw removal: AP view: 5.02 mm (SD ± 2.53). MO view: 1.32 mm (SD ± 2.58). Statistically significant decrease in both views (*p* ≤ 0.000).
Kaftadziev 2015 [21]	Retrospective Cohort	4	82	23	Patients with retained screws had significantly better outcomes compared to those with routinely removed screws. Improved outcomes were primarily attributed to the subset of patients with broken screws.	Patients with complications were excluded.	Three cortices: 66 patients (80%). Quadricortical fixation: 16 patients (20%). No correlation with clinical outcomes or screw fractures. Syndesmotic screw usage: single screw—71 patients (86%). Mean AOFAS scores: Intact screw (I): 83. Broken screw (B): 92.5. Removed screw (R): 85.5. Significant difference overall (*p* = 0.0496), mainly between groups I and B. No significant differences between groups I and R or B and R. VAS results: No significant differences in patient satisfaction (*p* = 0.34).
Kolodziej 2010 [22]	Retrospective Cohort	4	33	13	Removal of the syndesmotic screw did not significantly improve functional outcome.	Delayed wound healing: 2 patients. Skin changes around upper ankle and lower leg: 1 patient, indicative of post-thrombotic syndrome.	AOFAS score (mean): Screw removal group: 89 points (range: 80 to 100). Screw breakage group: 85 points (range: 80 to 98). Intact screw group: 87 points (range: 77 to 100). No significant differences between groups (*p* > 0.05).
Manjoo 2010 [31]	Retrospective Cohort	4	106	25	Intact screws: slightly worse functional outcomes compared to broken, loosened, or removed screws.	Indications for screw removal: Tenderness over screw prominence. Less than 10° of ankle dorsiflexion.	Tibiofibular clear space: Fractured, loose, or removed screws: 4.1 ± 0.2 mm. Intact screws: 3.1 ± 0.2 mm. *p* = 0.005. Medial clear space: Intact screws: 3.1 ± 0.2 mm. Broken, loose, or removed screws: 3.1 ± 0.1 mm. *p* = 0.9. Tibiofibular overlap: Intact screws: 7.0 ± 0.3 mm. Broken, loose, or removed screws: 6.9 ± 0.3 mm. *p* = 0.69.
Moon 2020 [23]	Retrospective Cohort	4	56	28	No difference in clinical outcomes between Screw removal group and screw retention group within three months. Screw breakage/loosening group and no issues group.	Group sizes: 9 (recurrence of diastasis) vs. 47 (no recurrence). AOFAS scores: 70.33 ± 6.22 (recurrence) vs. 76.50 ± 10.26 (no recurrence), *p* = 0.808. SF12-PCS scores: 49.85 ± 3.83 (recurrence) vs. 47.40 ± 8.01 (no recurrence), *p* = 0.948. SF12-MCS scores: 44.47 ± 4.47 (recurrence) vs. 46.97 ± 5.80 (no recurrence), *p* = 0.407. No significant differences based on screw size, number, or position.	Group sizes: 28 patients each (group A: screws removed in 3 months; group B: screws retained until 4 months). AOFAS scores: 75.10 ± 10.40 (group A) vs. 77.07 ± 10.60 (group B)—Not statistically significant. SF12-PCS scores: 45.78 ± 5.68 (group A) vs. 47.33 ± 5.83 (group B)—Not statistically significant. SF12-MCS scores: 48.45 ± 4.30 (group A) vs. 48.50 ± 10.04 (group B)—Not statistically significant.
Moore 2006 [24]	Randomized Controlled Trial	1	120	7	No difference in outcomes between retained and removed screws.	Two patients: late postoperative infections. Required hardware removal. Treated with intravenous antibiotics.	Group 1: Five patients (8%) had screw breakage. One required screw removal due to pain. Three had loss of reduction (noncompliance with weight-bearing restrictions before 6 weeks). Two had loss of fixation at the screw. Group 2: Four patients (7%) had broken screws. No loss of reduction with four cortices of fixation. Four patients (7%) had painful, prominent hardware, requiring screw removal.
Omrani 2019 [25]	Retrospective Cohort	4	60	18	Removing syndesmotic screws and allowing weight-bearing: potential benefit for anatomical alignment. Timely removal of screws: no improvement in foot functional outcomes.	No data.	18 patients (30%) with syndesmosis malreduction on initial postoperative CT. After screw removal (12 weeks), weight-bearing, and rehabilitation (4 weeks): 13 of 18 patients (72.2%) showed appropriate reduction on final CT scans.
Pogliacomi 2018 [26]	Retrospective Cohort	4	90	65	Syndesmotic screw removal: possibly unnecessary.	Patients with complications were excluded.	Group 1: 65 patients (72%), group 2: 25 patients (28%). Group 1: Screw removal after a mean of 7 weeks (range: 6–8 weeks). Group 2: 8 patients had broken screws; results similar to others. No significant differences in OMAS and AOFAS scores (*p* < 0.05). Tibiofibular clear space: similar in both groups, measured immediately and 1 year later (*p* < 0.05). All fractures healed after a mean of 3.5 months.
Sanda 2023 [32]	Retrospective Cohort	4	144	93	Patients who chose screw removal reported better satisfaction with mobility and daily activities. Removal group experienced reduced pain. Complications in the removal group: infection, loss of reduction. Potential negative impact on quality of life and mobility due to complications.	No data.	Postoperative screw removal improved mobility and daily activity performance. Reduced postoperative pain and anxiety in the screw removal group. No significant difference in overall quality of life between screw removal and conservative treatment groups.
Sanders 2021 [27]	Randomized Controlled Trial	1	152	85 (18 on demand)	On-demand screw removal had similar functional outcomes to routine removal. Routine removal group had significantly more complications (12 of 73) compared to on-demand removal group.	Significantly more complications in RR group (12/73) vs. ODR group (1/79) (*p* = 0.007). RR group complications: 5, wound dehiscence; 2, superficial SSI; 2, deep SSI; 1, diastasis after removal; 1, synovitis; 1, increase in stiffness. Four RR patients had syndesmotic fixation complications: 2 deep infections (1 causing flare-up post-removal, 1 leading to diastasis), 1 superficial SSI (wound dehiscence post-removal), 1 synovitis case (persisting post-removal).	Median OMAS at 12 months: 85 (IQR: 60–95) for RR; 80 (IQR: 65−100) for ODR. Noninferiority test: effect size within equivalent bounds of −10 to 10 scale points (*p* < 0.001). ODR not inferior to RR based on intention-to-treat and per-protocol analyses.
Schepers 2014 [28]	Retrospective Cohort	4	93	81	No difference in outcome scores among early, late, and no removal of the syndesmotic screw.	Six patients developed wound complications post-surgery. One 54-year-old male with a tri-malleolar Weber B fracture developed CRPS, AOFAS score: 19, OMAS score: 10, VAS score: 7, and single screw length: 36.6 mm.	AOFAS, OMAS, and VAS outcomes were not influenced by the number of engaged cortices or screw diameter. Two screws more frequently used in uni-malleolar fractures (27%) vs. bi- and tri-malleolar fractures (7%) (*p* = 0.033). Higher frequency of two screws in Weber C-type (24%) vs. B-type fractures (5%) (*p* = 0.007). Increased stiffness reported in OMAS subdomain for patients where screws were not removed or removed after 8 weeks (60%) compared to those removed within 8 weeks (29%) (*p* = 0.017). No significant effect on overall outcome between early and delayed removal groups.
Song 2014 [33]	Prospective, Prognostic Case Series	4	25	25	Initial malreduction rate: 36% following syndesmosis screw placement. 89% of malreduced syndesmoses corrected spontaneously after screw removal. Syndesmotic screw removal may assist in achieving final anatomical reduction in the distal tibiofibular joint.	Patient with continued malreduction despite screw removal: 21-year-old male. Fracture type: Weber B with medial malleolus fracture. Stabilization: Two 3.5 mm tricortical syndesmotic screws.	Initial postoperative CT: 9 patients (36%) with tibiofibular syndesmosis malreduction. Post-screw removal CT: 8 of 9 (89%) with initial malreduction showed adequate reduction. Statistical significance: Significant reduction in malreduction (t = 3.333, *p* < 0.001). Malreduction rates: Initial rate of 36% (9/25), post-screw removal rate of 4% (1/25).
Tucker 2013 [29]	Retrospective Cohort	4	63	43	Retention of screw: No significant reduction in functional capacity. Cost-effectiveness: Provides added cost-effectiveness.	No data.	Mean OMAS scores—Retained group: 81.5 ± 19.3; removed group: 75 ± 12.9 (*p* = 0.107). Functional scores: Higher in retained group across OMAS domains. Pain levels: Lower in retained group. Adjusted for gender: Significant results (*p* = 0.046).
Weening 2005 [30]	Retrospective Cohort	4	51	30	OMAS and SMFA scores: no significant difference between removed and retained screws.	Malreduction causes: Fibular fracture malreduction in 2 patients. Tibiofibular misalignment in 6 patients.	Multivariable model: no significance for age, number of cortices, medial malleolar fracture, ankle dislocation, appropriateness of screw fixation, or screw removal. Medial malleolar fracture: no significant difference in SMFA-functional index (12.7 ± 13.6 vs. 9.9 ± 14.9, *p* = 0.64) and OMAS (73.5 ± 25.9 vs. 70.0 ± 20.5, *p* = 0.82). Ankle dislocation: trend toward decreased function (12.9 ± 8.9 vs. 8.1 ± 7.2, *p* = 0.09). Screw insertion appropriateness: no significant difference in SMFA-functional index (9.1 ± 14.3 vs. 15.1 ± 14.1, *p* = 0.68).
Yang 2021 [34]	Retrospective Cohort	4	113	113	Longer screw retention: might be necessary to prevent syndesmotic diastasis recurrence in tri-malleolar fractures without posterior malleolar fixation. Posterior malleolar fragment: even small fragments should be considered a risk factor for recurrent syndesmotic instability.	Superficial infection: two patients, one in Group I and one in Group II.	Functional outcomes: no significant difference among groups. Recurrence rates: Group I: 10.6%. Group II: 20.9%. Group III: 8.7%. Recurrence rate significance: not statistically significant (*p* = 0.264). Tibiofibular clear space changes: greater interval change in group II (*p* = 0.028).

## 4. Discussion

A comprehensive review of the existing literature did not reveal notable differences in functional outcomes when syndesmotic screws are removed following tibiofibular syndesmosis stabilization. This indicates that the removal of these screws does not significantly impact the overall functional recovery of patients, as supported by current research.

Several studies have examined the relationship between syndesmotic screw removal and functional outcomes post-stabilization. Despite variations in methodology and sample sizes, a consistent trend shows no substantial change in functional recovery after screw removal.

This suggests insufficient justification for routinely removing syndesmotic screws. Findings from five RCTs also indicated no significant functional differences between removing the screws and leaving them in place.

### 4.1. Retain and Remove Outcomes

Thirteen retrospective studies involving a total of 855 patients did not show any significant statistical differences in ankle joint function between patients who had syndesmotic screws removed and those who retained their screws after tibiofibular syndesmosis fixation. Moon et al. conducted a study to determine whether the removal of syndesmotic screws before weight-bearing ambulation impacts clinical outcomes in patients with distal tibiofibular syndesmosis injuries. The study included 56 patients divided into groups based on whether their screws were removed (n = 28) or retained (n = 28) and whether they experienced recurrence of diastasis (n = 9) or not (n = 47). Results showed no significant differences in the American Orthopaedic Foot and Ankle Society scale (AOFAS) and Short-Form Health Survey-12 (SF-12) between screw-removed and -retained groups. However, the recurrence of diastasis was significantly higher in the screw-removed group (*p* = 0.025). The study concluded that removing syndesmotic screws before weight-bearing is unnecessary, as it does not influence clinical outcomes [23]. Jordan et al. aimed to evaluate the radiographic changes in tibiofibular position and the ankle mortise after the removal of trans-syndesmotic screws in patients with displaced ankle fractures. The retrospective study included 86 patients who underwent open reduction with syndesmosis screw stabilization. The key findings indicated a significant increase in tibiofibular clear space (from 4.63 mm to 5.41 mm) and a decrease in tibiofibular overlap (from 5.83 mm to 5.02 mm) post-screw removal, suggesting a high correlation of loss of syndesmotic integrity. Despite these radiographic changes, the medial clear space remained relatively stable, indicating that while there is a common occurrence of tibiofibular diastasis upon screw removal, the ankle mortise maintains its stability [37]. 

Kaftandziev et al. stated that the aim of their study was to compare clinical outcomes between patients who retained the syndesmosis screw and those who had it removed following the open reduction and internal fixation of malleolar fractures associated with syndesmosis disruption. The study included patients treated from January 2011 to December 2012, excluding those with incomplete data or specific postoperative complications. The findings showed no statistically significant difference in clinical outcomes between patients with the screw retained and those with the screw removed. However, patients with a syndesmotic screw fracture had better clinical outcomes. Routine removal of the syndesmosis screw is not recommended based on these results [21]. Hamid et al. demonstrated in their study the comparison of clinical and radiological outcomes in patients with Weber B or C ankle fractures and associated syndesmosis injuries, focusing on the condition of the syndesmosis screw (intact, broken, or removed). The study included 52 patients out of a possible 142 who met the inclusion criteria and returned for assessment at least one year post-surgery. Of these, 27 had intact screws, 10 had broken screws, and 15 had undergone elective screw removal. The findings revealed that the mean AOFAS scores were 83.07 in the intact screw group, 92.40 in the broken screw group, and 85.80 in the removed screw group. Interestingly, patients with broken screws exhibited the best clinical outcomes. The study concluded that there was no significant difference in outcomes between patients with intact and removed screws and suggested against the routine removal of syndesmosis screws, whether intact or broken [17]. Hsu et al. aimed to investigate the outcomes of syndesmotic screw fixation in the treatment of syndesmotic diastasis. They conducted a retrospective study on 52 adult patients treated for syndesmotic diastasis with a trans-syndesmotic cancellous screw, following strict inclusion criteria and excluding patients with pilon fractures or insufficient follow-up. Patients were grouped based on the timing of syndesmotic screw removal: six weeks, three months, and an average of nine months. The study compared recurrence rates of syndesmotic diastasis, incidence of screw breakage, and ankle function among these groups. The findings revealed that syndesmotic diastasis recurrence rates were 15.8% in the six-week removal group, 15.0% in the three-month group, and 0% in the nine-month group, though this difference was not statistically significant. Screw breakage occurred in 15.0% of patients within three months and 15.4% beyond three months, with no breakages in the six-week group. Overall, 82.7% of patients had satisfactory outcomes, and ankle function did not significantly differ among the groups, regardless of screw breakage or syndesmotic diastasis recurrence. The study concluded that while early removal of the syndesmotic screw might prevent its breakage, it could increase the risk of syndesmotic diastasis recurrence [39]. One possible explanation for the finding that timing did not affect functional outcomes could be the inherent stability provided by the syndesmotic fixation itself. Syndesmotic screws are primarily used to maintain proper alignment and stability of the tibiofibular syndesmosis during the initial phases of healing following injury. Once the ligaments have sufficiently healed and the syndesmosis has regained stability, the necessity of the screws for maintaining alignment diminishes.

Furthermore, the absence of a significant impact of timing on functional outcomes may also be attributed to the body’s natural healing processes. Over time, the surrounding soft tissues, ligaments, and muscles adapt and strengthen, contributing to the overall stability of the ankle joint. This inherent healing capacity may compensate for any minor disruptions caused by the timing of syndesmotic screw removal.

Additionally, it is important to consider the rehabilitation protocol employed post-surgery. Regardless of the timing of screw removal, patients typically undergo structured rehabilitation programs aimed at restoring strength, flexibility, and function. These rehabilitation efforts likely play a crucial role in facilitating functional recovery, potentially mitigating any differences attributable to the timing of screw removal.

Two retrospective studies examining 250 patients show slightly worse results in patients who had syndesmotic screws left in place. 

Sanda et al. aimed to evaluate whether the removal of syndesmotic screws post distal tibiofibular diastasis repair improves patient outcomes in terms of quality of life, mobility, and daily living activities, and whether it is a cost-effective solution. The study included patients with uni-malleolar or bi-malleolar ankle fractures, who were evaluated using standardized questionnaires approximately two months post-surgery. Out of the participants, 93 had their screws removed, while 51 retained them. The results showed that patients with screw removal reported better mobility (7.8 vs. 6.7) and ability to perform daily activities (8.1 vs. 6.5) and experienced less pain (5.3 vs. 6.8). Additionally, these patients had higher scores on the SF 6 physical domain (55.9 vs. 53.3) and lower anxiety levels (5.8 vs. 7.3). However, overall quality of life and willingness to recommend the treatment did not significantly differ between the groups. Thus, screw removal post-surgery enhances specific aspects of recovery, but the overall quality of life remains comparable [32]. Yang et al. intended to assess the outcomes of fixation for bi-malleolar and tri-malleolar ankle fractures with syndesmotic injury, particularly assessing the effects of early versus delayed removal of syndesmotic screws. The study focused on whether removing these screws at 6 to 8 weeks or at 3 months postoperatively offers more benefits. Patients who underwent open reduction and internal fixation for these fractures between January 2013 and December 2017 were analyzed, with a minimum follow-up of 24 months. Patients were categorized into three groups based on the timing of syndesmotic screw removal: group I (bi-malleolar fractures with removal at 6 to 8 weeks), group II (tri-malleolar fractures with removal at 6 to 8 weeks), and group III (tri-malleolar fractures with removal at 3 months). The study included 113 patients. Results indicated no significant difference in ankle functional outcomes among the groups. However, recurrence of syndesmotic instability was observed to be higher in group II (20.9%) compared to group I (10.6%) and group III (8.7%). Despite the lack of statistical significance in recurrence rates, group II showed a significant interval change in tibiofibular clear space compared to the other groups, suggesting potential benefits of delayed screw removal [34].

Ibrahim et al. studied the incidence and predictors of intraosseous screw breakage in syndesmotic stabilization and its association with implant removal due to pain. They retrospectively reviewed patients at a level 1 trauma center from 2011 to 2018, identifying 43 patients with 58 broken screws. The study aimed to determine the incidence of intraosseous screw breakage, identify clinical and radiographic predictors, and assess if IO breakage was associated with higher rates of painful implant removal. Findings showed that 74.4% of screw breakages occurred intraosseously, significantly linked with subsequent removal due to pain (*p* = 0.034). Only screw height from the tibial plafond significantly predicted breakage location, with screws placed 20 mm or more from the tibiotalar joint being less likely to break intraosseously (OR: 0.818, *p* = 0.002). The study highlighted the clinical importance of screw placement in preventing painful complication [38].

### 4.2. Complications

Two retrospective studies involving 368 patients demonstrate that reoperation, such as routine removal of syndesmotic screws, is associated with an increased risk for the patient. Infectious complications are primarily reported, with the incidence of complications ranging from 1.4% to 6%.

### 4.3. Limitation

The present study also has several limitations. Primarily, this review included a limited number of RCTs, resulting in weaker evidence. Furthermore, conducting a meta-analysis would be challenging due to the diversity of data analyzed across the studies mentioned. To establish a unified consensus based on a reliable meta-analysis, additional replicable RCTs would be required. Although an RCT protocol was designed by Dingmans et al. in 2018, there remains an insufficient number of such studies available [40].

## 5. Conclusions

The current literature does not provide definitive evidence supporting the superiority of routinely removing syndesmotic screws over retaining them. Considering the heightened risk of complications and the additional financial burden associated with routine removal, it is advisable not to perform this procedure unless specifically indicated. Further RCTs are required to determine whether there are any differences in functional and clinical outcomes between patients who undergo syndesmotic screw removal and those who retain them following tibiofibular syndesmosis fixation. The routine versus on-demand removal of the syndesmotic screw (RODEO) trial, an international protocol for RCTs, serves as an example of such a study aimed at assessing the efficacy of routine syndesmotic screw removal [40].

## Figures and Tables

**Table 1:** Characteristics of studies evaluating the routine removal of syndesmotic screws.

Author, Year of the Study	Type of Study	Strength of Evidence	Number of Patients	Key Findings
Jacobsen 1994 [10]	Retrospective	4	66	About 75% of patients reported improvement after removal.
Kaye 1989 [11]	Retrospective	4	30	No screw broke prior to removal. Calcification of the interosseous membrane observed in 6 patients. Distal tibiofibular synostosis developed in 4 patients. Transfixation screws provided satisfactory stability of the syndesmosis. Permitted stable healing of the interosseous membrane and distal ligaments after ankle fracture.

## Data Availability

The data presented in this study are available on request from the corresponding author (B.G.W.).
